# A Blockchain of Things System for Managing Handcrafted Products in a Cultural Industry

**DOI:** 10.3390/s24227384

**Published:** 2024-11-20

**Authors:** Youssef Aounzou, Fahd Kalloubi, Abdelhak Boulaalam

**Affiliations:** 1 LSI Laboratory, National Schools of Applied Sciences, Sidi Mohamed Ben Abdellah University, Fez 30000, Morocco; abdelhak.boulaalam@usmba.ac.ma; 2 Vanguard Center, Mohamed VI Polytechnic University, Benguerir 43150, Morocco; kalloubi.f@ucd.ac.ma; 3 National School of Applied Sciences, Chouaib Doukkali University, El Jadida 24002, Morocco

**Keywords:** Internet of Things, blockchain technology, blockchain of things, handicraft industry, smart contract, anti-counterfeits technology

## Abstract

The handicraft sector is often seen as a symbol of a country’s cultural identity, as it relies on specialized traditional techniques, skills, and knowledge that are often passed down through generations. As a result, ensuring the authenticity and integrity of this creative value has become a significant challenge, especially with the growth of counterfeiting techniques in this industry. Thus, integrating digital technologies into such sectors offers numerous operational benefits such as transparency, decentralization, data security, and authenticity needs. This paper presents an innovative approach for the handicraft sector, which exploits blockchain technology and the Internet of Things to guarantee the authenticity of cultural heritage. Through experimental evaluations comparing the decentralized blockchain-based system with traditional centralized methods using key metrics such as response time and transactions per second, this study reveals significant results. The statistical analysis reveals that the decentralized approach improves performance in terms of response times for verification and addition processes compared to the centralized system. Specifically, verification is approximately 4.66 times faster and addition is approximately 4.30 times faster in a decentralized system. However, transaction latency in the decentralized approach is approximately 38.21% higher than in the centralized system.

## 1. Introduction

### 1.1. Motivations

Traditional knowledge has always been considered a vital cultural, social, and historical asset for the communities that retain it. This cultural identity is exhibited through the skills used, technologies employed, and especially by handicrafts produced by craftsmen. This kind of product attracts tourists to visit countries known for this industry, consequently improving the cultural tourism industry. According to the UNWTO organization [1], tourism products are defined as a mixture of tangible and intangible elements, including natural and cultural resources, attractions, facilities, services, and activities. In the cultural tourism industry, these elements are combined to provide a great experience to tourists. Furthermore, this kind of product has taken an interesting place in the market due to its positive cultural identity compared to other products of the same kind.

However, this increased attention has also led to an increase in the imitation of such products, making it increasingly difficult to distinguish between genuine and counterfeit items. This difficulty is further compounded by the prevalence of mass-produced counterfeit products that bear a striking resemblance to the originals.

The proliferation of counterfeits poses a major obstacle in various fields, not only affecting legitimate producers but also leading to consumer distrust and financial losses. Genuine brand owners invest significantly in product design, marketing, and manufacturing, while counterfeiters exploit brand names without incurring these costs. Extensive research has delved into the effects of counterfeiting across diverse domains. For example, [2] examines how the infringement of intellectual property rights impacts the innovation performance of enterprises in China.

This underscores the urgent need to ensure that products and creations maintain their true origin and authenticity. By protecting this fundamental value, not only is the integrity of products and ideas preserved, but also consumer trust is strengthened, fostering innovation and creativity in various sectors. However, the preservation of authenticity can have a dual impact by enhancing both the appeal of heritage destinations and the efficiency of their management. The authors in [3] highlight the importance of authenticity and engagement in the context of luxury hotels. It focuses on the guest experience in this specific environment, exploring how authenticity significantly contributes to the creation of exceptional hotel experiences. Thus, preserving authenticity is essential to maintain the cultural identity and economic viability of handcrafted products.

### 1.2. Innovations

To address the challenge of counterfeiting, this paper proposes a decentralized system leveraging blockchain and IoT technologies. IoT is an innovative technology connecting the physical and digital worlds, with a layered architecture spanning perception, processing, and application layers. Devices like sensors, RFID tags, and controllers collect data at the perception layer, interacting with the environment through actuators. These data are then transmitted to the communications layer, connecting devices to IoT gateways via protocols like Bluetooth and NFC. The application layer utilizes the data collected and processed by the lower layers, presenting it to end users through widely adopted protocols. Nonetheless, the implementation of IoT has posed challenges, notably regarding the security and privacy of IoT devices. However, these challenges can be mitigated by technologies such as Blockchain, known for transparently and securely storing and transmitting information in linked, immutable blocks. These technologies complement each other to increase transparency, traceability, and authenticity verification throughout the product lifecycle. By combining blockchain and IoT, the proposed system aims to create a flexible and efficient tracing mechanism to validate product integrity and origin. Despite challenges such as security and scalability, these technologies hold promise in combating counterfeiting in the handicraft sector.

### 1.3. Contributions

The main contributions of this paper include the following:

- Proposing a decentralized system for authenticating handcrafted products.
- Integrating blockchain and IoT technologies to combat counterfeiting in the handicraft industry.
- Conducting extensive experiments to demonstrate the superiority of the decentralized system over centralized architectures.
- Performing load tests to evaluate the system’s effectiveness in terms of latency and response time.

### 1.4. Manuscript Structure

The remainder of this paper is organized as follows. Section 2 reviews the existing literature and discusses relevant studies on authenticity preservation. Section 3 provides a detailed explanation of the proposed approach, including the integration of blockchain and IoT. Section 4 presents the results of experiments and initiates essential discussions. Section 5 discusses the impact of the approach and applications. Section 6 discusses the challenges of the approach. Finally, Section 7 offers a conclusion along with suggestions for future research directions. Table 1 lists the notations used throughout this paper.

## 2. Related Work

A multitude of research endeavors have been undertaken to propose innovative solutions aimed at preserving the authenticity of products and countering the pervasive issue of counterfeiting. This section offers an extensive overview of the diverse studies conducted in various domains, each leveraging distinct information technologies, such as blockchain and IoT, to tackle this multifaceted problem.

Counterfeiting represents a formidable challenge for the manufacturing sector, driving a proliferation of research efforts directed at comprehending the implications and ramifications of this important issue. These studies are focused on elucidating the consequences of counterfeiting, comprehending its far-reaching impact, and emphasizing the imperative need for robust anti-counterfeiting measures. These strategies, as articulated in these studies, can be implemented at various stages of production.

For example, within the context of the Vietnamese industry’s intricate supply chain, ref. [4] advocates for the adoption of RFID technology to substantiate the authenticity of products. The deployment of RFID technology is projected to foster sustainability by enhancing traceability, optimizing stock management, and mitigating wastage and inefficiencies. Nonetheless, it is vital to acknowledge certain drawbacks associated with this solution, particularly its vulnerability in packaging and labeling methods, rendering them susceptible to easy replication and counterfeiting. Consequently, counterfeiters can exploit this vulnerability to reproduce authentic product labels when deemed profitable.

The adoption of IoT technology has also demonstrated its efficacy in addressing the multifaceted challenges related to product identification and tracking. Within the craft sector, several works have introduced IoT-based systems to guarantee the identification of handicraft products [5,6]. Moreover, the integration of IoT has proven instrumental not only in product identification and authentication but also in PLM across diverse sectors. Numerous research studies have underscored the importance of integrating IoT within PLM [7,8], with a specific focus on manufacturing processes, product development, the and overall enhancement of business performance. This integration provides substantial advantages, including real-time data collection, analysis, and communication, thereby enhancing visibility and control over various facets of production. Additionally, it fosters collaboration and information sharing among stakeholders, thereby infusing PLM with the advantages of improved operational efficiency, cost reduction, and an accelerated time to market [9]. The data generated by IoT also empowers data-driven decision-making, enabling organizations to adapt to market demands and enhance overall performance. However, it is imperative to address and resolve the challenges posed by the adoption of IoT in terms of data security, interoperability, and standardization by deploying complementary technologies. Blockchain technology has garnered considerable interest across a spectrum of industries due to its manifold benefits. Presently, it is being harnessed in diverse fields to address persistent issues, including the management of intellectual property lifecycles, as comprehensively detailed in [10,11,12,13]. Furthermore, blockchain is being deployed to combat counterfeiting, with the authors in reference [14] providing insights into a decentralized blockchain-based ecosystem, posited as an effective solution to thwart counterfeiting and ensure product traceability throughout the entire manufacturing process.

Beyond manufacturing, blockchain technology has found applications in diverse industries to address challenges and deliver a multitude of benefits. In the smart tourism industry [15], blockchain has been employed to establish a secure and decentralized connection between attractions and visitors. Similarly, the diamond industry has embraced blockchain to address concerns surrounding authenticity, traceability, third-party verification, and transaction reliability, potentially elevating its status as an investment asset [16].

The merging of blockchain and IoT technologies is proving to be a robust and effective strategy to face the challenges raised in different areas, offering significant benefits across various sectors, including smart cities and Industry 4.0. Numerous studies, such as those by [17,18], have demonstrated how this integrated approach can create secure and transparent systems, enabling real-time tracking and ensuring authenticity.

In the field of smart cities, several innovative approaches integrating blockchain and IoT are proposed [19], targeting various sectors such as smart grids, ITSs, and healthcare 5.0. These initiatives illustrate the significant potential of blockchain combined with IoT to transform each application domain while highlighting the open research challenges that remain to be addressed.

For smart grids, a system detailed in reference [19] proposes a secure peer-to-peer (P2P) energy trading architecture. This configuration allows users to trade energy transparently and securely, without the need for a centralized intermediary, thanks to the blockchain that records all energy transactions, increasing transparency and reducing the risk of fraud. This system promotes more flexible and distributed management of energy production and consumption, which could improve the efficiency of energy distribution and encourage the use of renewable energies. The architecture is divided into three main layers: the energy generation and distribution layer, the communication layer, and the consumer–prosumer layer.

In the transport sector, a model integrating unmanned aerial vehicles with blockchain to enhance intelligent transport systems is proposed in reference [19]. UAVs collect traffic data in real-time, while the blockchain guarantees the security and integrity of these data, optimizing traffic flows, improving road safety, and reducing congestion. The proposed architecture consists of three interconnected logical layers: the application layer, the communication layer, and the blockchain layer, bringing intelligence, flexibility, trust, security, reliability, and efficiency to the ITS communication system in a 6G environment.

As detailed in many studies [19,20], the intelligent healthcare system uses blockchain to secure and immutably manage medical records, ensuring that only authorized individuals have access to them. The system proposed in reference [19] supports telemedicine and electronic prescriptions, improving access and the quality of healthcare while protecting patient confidentiality. Adapted to 6G communication networks, the architecture includes applications, such as remote monitoring and telemedicine, and also responds to the needs generated by the COVID-19 pandemic, consisting of three interconnected logical layers: the healthcare application layer, the communication layer, and the blockchain layer, reinforcing the flexibility, trust, security, confidentiality, reliability, and efficiency of the communication system in the healthcare sector.

In the specific context of the textile industry, this combination of blockchain and IoT has led to the development of advanced traceability systems. Such systems ensure the integrity of supply chains and enhance the traceability of materials from source to store [21]. This not only helps in maintaining the quality of the products but also fosters trust among consumers by providing them with transparent product histories.

In the Industry 4.0 field, blockchain technology, coupled with IoT, is becoming a cornerstone, offering transformative solutions for enhancing transparency, security, and efficiency across various manufacturing sectors. For instance, a comprehensive survey by experts in the field delves into how blockchain can revolutionize sustainable manufacturing and PLM within Industry 4.0 frameworks [22]. This integration enables a more sustainable approach to production processes by ensuring product traceability and improving lifecycle assessments.

Furthermore, a thorough review encapsulates various blockchain applications across Industry 4.0, highlighting significant improvements in interoperability and data sharing between complex industrial systems [23]. The synergy between blockchain and IoT technologies facilitates a new level of communication and data exchange, with a variety of areas showing significant potential, but it also raises inherent problems beyond those addressed by the IoT. These include vulnerabilities to majority attacks and double-spending issues. These issues with the mass adoption of blockchain technology compromise the integrity and fundamental trust model of blockchain systems, potentially challenging the reliability of transactions and the security of data.

To deal with these vulnerabilities, the integration of data analytics into blockchain-secured data becomes crucial. As detailed in reference [24], this approach strengthens the reliability and security of blockchain systems, using advanced data analysis techniques to detect and prevent malicious or fraudulent activity.

Similarly, the role of heuristic/evolutionary search optimization AI-based algorithms in the handicraft industry, such as the gravitational search algorithm [25] and Inclined Planes System Optimization [26], has been significant in optimizing various aspects of handicraft production and distribution. These algorithms contribute to the development of more efficient and cost-effective handicraft manufacturing processes, as well as improved decision-making capabilities in the handicraft industry.

Both the integration of data analytics into blockchain-secured data and the use of heuristic/evolutionary search optimization AI-based algorithms represent promising areas of research and development to meet the current and future challenges associated with the large-scale adoption of these technologies. The synergy between blockchain and data analysis, and the application of AI-based optimization algorithms in various industries, offers a more robust and reliable solution for applications requiring a high level of integrity and trust in transactions and data, such as financial systems, supply chains, and land registries.

Table 2 provides a summary of the advantages and disadvantages of using these technologies in different contexts, according to the sources provided.

## 3. Proposed System

In this section, we present the structure of our proposed platform based on blockchain and IoT, which aims to address the issue of product counterfeiting in the handicraft sector. The proposed platform offers an innovative solution to combat fraudulent activities by leveraging the inherent capabilities of blockchain technology. We dissect the intricate details of the platform’s system architecture, describing its robust framework and components that work in tandem to ensure transparency, traceability, and authenticity throughout the supply chain.

### 3.1. System Architecture

**Figure 1:**
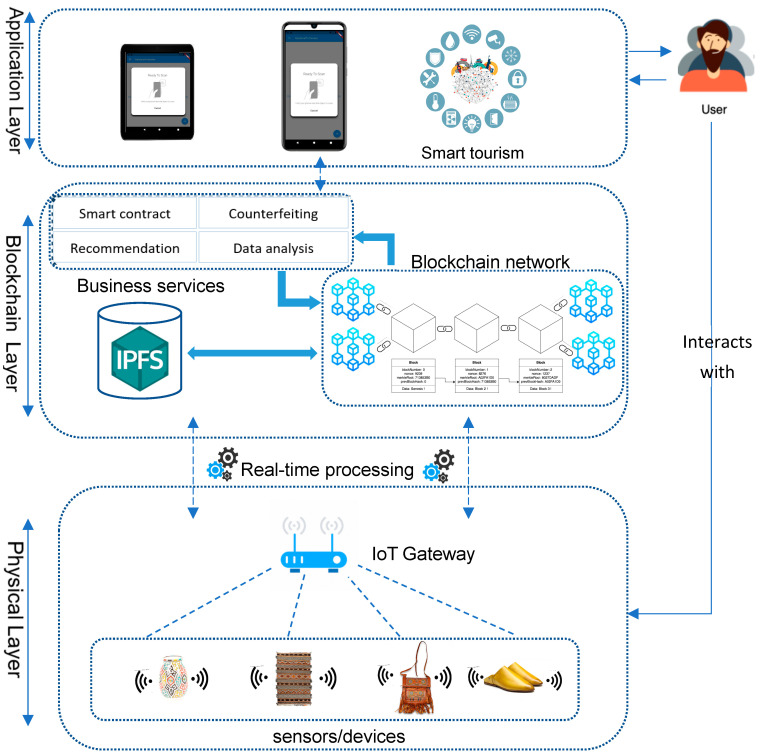
System overview.

The proposed architecture for an intelligent craft environment, depicted in Figure 1, consists of three layers: physical, blockchain, and application. Each layer has distinct goals and provides various features. In the following sections, we will provide a concise overview of each layer of the proposed architecture.

#### 3.1.1. Physical Layer

The physical layer serves as a sensing layer that contains several types of IoT devices placed in different locations, such as on items, in retail stores, and in other locations to collect real-time data about the products that could be used to guarantee the digital identity. The transmission of collected data is ensured by a gateway, which allows the reliable, efficient, and coordinated connection between the physical layer and other layers, thereby guaranteeing the retrieval and transfer of obtained data. After the pre-processing tasks of analysis and processing of these collected data, they are then transmitted to the persistence layer to provide specific services to users. In this way, product consultation or sales operations can be stored and tracked at every stage. The use of the digital representation of physical products allows the monitoring of material flows for better transportation management and accurate risk management.

The physical layer of the platform uses a range of wireless technologies to establish connections with various objects in the system. These technologies include NFC, Bluetooth, Wi-Fi, ZigBee, global positioning systems (GPS), sensor networks, and LoRaWAN. Each of these wireless technologies possesses unique characteristics and finds application in specific areas. NFC enables short-range communication for secure data exchange.

The using of these diverse wireless technologies caters to different requirements and enables seamless connectivity within the system.

In the context of WSNs, the LEACH is a popular routing technique that supports fault tolerance, load balancing, and reliable communication and prolongs the network lifetime.

However, the random selection of CHs in LEACH protocols results in poor performance due to the faster rate of energy depletion at CHs. The dynamic selection of CHs based on a heuristic approach can minimize energy consumption at CHs and enhance the network lifetime.

A novel metaheuristic for cluster head selection, called ARSH-FATI, has been developed and integrated with a heuristic called ranked-based clustering [27]. This approach aims to optimize the selection of CHs and enhance energy efficiency in WSNs. The ARSH-FATI-based cluster head selection algorithm incorporates a heuristic that considers the sink distance to the CH and the residual energy at the sensor node to define the fitness function. The optimal values of the fitness function provide an efficient CH selection and cost-effective routing, ultimately maximizing the network lifetime of WSNs.

Moreover, recent advancements in IoT-based healthcare emphasize the critical need for energy-efficient data processing on edge devices. The study presented in “Energy-Aware Scheduling of Streaming Applications on Edge-Devices in IoT-Based Healthcare” [28] explores efficient scheduling strategies that can be synergistically applied to WSNs, enhancing both the computational and communication paradigms at the edge of healthcare networks.

The integration of efficient cluster head selection algorithms, such as ARSH-FATI-CHS, with wireless technologies in the physical layer can significantly enhance the performance and energy efficiency of WSNs and IoT systems.

#### 3.1.2. Blockchain Layer

**Figure 2:**
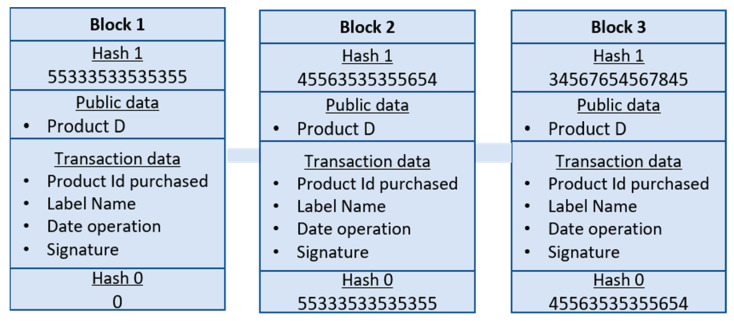
Blockchain data sample.

The persistence layer, also known as the blockchain layer, serves as the fundamental component of the system and relies on essential modules that organize the core services of the blockchain. Its primary objective is to ensure seamless data collection, transmission, and communication operations while safeguarding the integrity of the data against counterfeiting. This layer functions as a distributed ledger, responsible for storing transactional and operational data. The ledger comprises a series of interconnected blocks, linked together using cryptographic hash functions, which are integral to the blockchain system. Each valid purchase transaction made by a customer for a tagged product is recorded in blocks and interconnected to form a chain, as depicted in Figure 2; when a transaction occurs, new blocks are added to the network. These blocks contain essential information such as the hash of the current block, the hash of the previous block, and transaction details, including the product ID, tag name, transaction date, and signature, all without any human intervention. The blockchain platform we use in our system is Ethereum, which currently uses Proof of Stake (PoS) as its consensus mechanism. We aim to integrate the Proof of Authenticity into our Ethereum application, thereby taking advantage of its considerable benefits. To achieve this goal, we have developed a smart contract specifically designed to handle block validation. In this smart contract, we have established the guidelines governing block validation and the procedures that validators must follow to demonstrate their authenticity and legitimacy within the network.

Once the system receives the transactions to be validated, these transactions are grouped into blocks and then sent to a dedicated smart contract. This smart contract takes on the responsibility of initiating the verification process using the PoA protocol. After the validation is completed, the PoA protocol triggers an update of the records within the blockchain, allowing for the publication of blocks containing valid transactions across the entire network. This sequence ensures that transactions undergo a validation process focused on authenticity through the PoA algorithm, ensuring their integrity and availability to all network participants. Algorithm 1 describes this multi-step process for using the Proof of Authenticity algorithm. All symbols used in algorithms are described in Table 3. The process begins with the receiving of a list of these nodes, denoted as Nnv (Nnv1, Nnv2, Nnv3, …), for authentication. Each node undergoes a thorough verification by a trusted node Nt, which utilizes the ECDSA algorithm to generate and verify public and private key pairs, securing data transmission in the process. During the verification, if Nt.verify(i) returns true, the node is deemed authentic and is added to Nv, a list of verified and trustworthy nodes. If the verification fails, the node remains in Nnv or is subject to further security protocols, potentially including isolation or additional diagnostics. This continuous process of verification and categorization enhances the reliability and integrity of the network by ensuring that only validated nodes partake in network activities, effectively safeguarding the system against security breaches and maintaining operational resilience. The validated nodes are then compiled into a list of valid blocks for integration into the blockchain, completing the Proof of Authentication cycle and reinforcing the network’s defense mechanisms.

To register products in the blockchain network, the organization sends a set of transactions to be verified, which are grouped in a node. As shown in Algorithm 2, it is initiated with a set of transactions represented as *Nnv* = *T*1, *T*2, *T*3; the algorithm processes each transaction to extract and verify product identifiers. Each product ID derived from a transaction is checked against existing blockchain entries to determine uniqueness via the method *c*ℎ*eckExistingProduct*- *InBlockC*ℎ*ain*. If the product is not already recorded in the blockchain (*Sp* returns false), a new node Ni is created using *createNodeForProduct*(*Pi*), and the product undergoes Proof of Authenticity processing *ExecutePoA*ℎ*Processing*(*Ni*), securing its registry on the blockchain. If verification finds that the product already exists or fails any subsequent checks, the transaction is rejected, marked by the Boolean output *S* set to false. Successfully registered products, on the other hand, result in *S* being set to true, indicating a successful addition to the blockchain. This process not only ensures that each product is uniquely logged but also strengthens the network’s integrity by preventing duplications. Upon successful registration, the blockchain generates a certificate that identifies the user using their private key along with a Boolean value indicating the processing status. This certificate is then transmitted to the user.
**Algorithm 1.** Execution process of the PoAh

1: **function** PROCESSPOAH (*Nnv*)
2: **Input:** Nnv = Nnv1,Nnv2,Nnv2,...▷ a set of invalided nodes in the network
3: Nt: a trusted node that verifies the authenticity of invalid blocks.
4: **Output**: Nv = Nv1,Nv2,Nv3,...  
▷ a set of validated nodes in the network.

5: **for each** node *i* in *Nnv*
**do**

6:       
**if**
*Nt.verify(i*) is true **then**

7:             *Nv*←
    *Ni*
8: 
      
**else if**
*Nt.verify(i)* is false **then**

9: 
            
*Nnv*
←
    
*Ni*
10: 
      
**end if**
11: **end for**
12: Return *Nv*

13: 
**end function**


The business layer also encompasses all the essential services required for the functionality of the proposed system, such as anti-counterfeiting, smart contracts, recommendations, and real-time tracking services. It offers a set of functionalities to external users of the system, such as the counterfeit verification service, which aims to allow users to search the network using a smart contract detailed in Algorithm 3 to verify the status of a product. The operational flow of Algorithm 3 works as follows. It starts by extracting a product ID from the transaction using the function getProductIdFromTransaction(T). The algorithm then retrieves a list of validated products stored on the blockchain through getValidatedProductFrom- Blockchain. It iterates over each product in this list to check for a match with the product ID derived from the transaction. If a matching product ID (Pi = P1) is found within the blockchain’s validated entries, the algorithm returns true, confirming the product’s legitimacy. Conversely, if no match is found (Pi != P1), the result is false, indicating a potential counterfeit. This methodical verification ensures that only products verified as genuine are acknowledged by the network, significantly enhancing the security measures against counterfeiting. By integrating this verification process, the blockchain network provides a transparent and reliable service for confirming product authenticity, crucial for maintaining trust and integrity in markets prone to counterfeit challenges.
**Algorithm 2.** Product Registration Process

1: **function** REGISTRATIONPROCESS(Nnv)
**2: 
Input: Nnv = T1,T2,T3...** 
▷
a set of transactions

3: **Output**: S Boolean 
▷
State of registration processing of the node.

4:        
**for each** transaction *i* in *Nnv*
**do**

5: *  *
    
*Pi = getProductIdFromTransaction(Ti)*

*6: *
      
*Sp = checkExistingProductInBlockChain(Pi)*

7: 
      
**if**
*Sp* is false **then**

8: 
            
*Ni = createNodeForProduct(Pi)*

*9: *
              
*ExecutePoAhProcessing(Ni)*

*10: *            *S*
← *    true*

11: 
      
**else if** Nt.veri f y(i) is true **then**

12:
                    
*S*
← *    false*

13:
            
**end if**

14:
        
**end for**

15: Return S

16: **end function**


**Figure 3:**
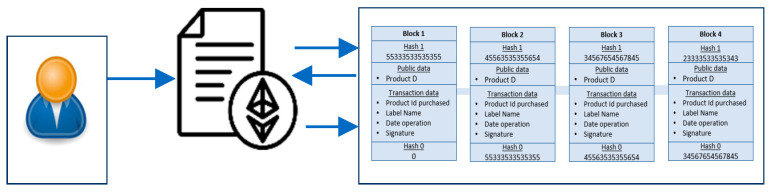
Product verification workflow.

As described previously, the suggested system uses several smart contracts to allow end users to interact independently on a decentralized blockchain network. This functionality allows customers and external applications to access the registry according to their needs using the corresponding smart contracts. The complete process of product verification using the Check Counterfeiting Product smart contract is illustrated in Figure 3. When the user approaches the product with their phone to check its status, they interact directly with the verification module. This module subsequently initiates a verification request to the blockchain, including the relevant product information. Upon receiving the request, the blockchain processes it and sends the response back to the user. In most cases, these smart contracts on the blockchain are written in Solidity, which is a programming language capable of generating transactions, executing decisions, and storing data automatically.

To ensure real-time data persistence in our system, we have integrated the IPFS storage solution into the blockchain layer; this decision brings a myriad of invaluable benefits to our platform. The IPFS, a technology designed to overcome the difficulties of conventional data storage, offers significant advantages.

First and foremost, the IPFS decentralizes storage, eliminating costly dependence on centralized servers. This decentralization also enhances data security, which is no longer vulnerable to a single source of failure. Also, the IPFS ensures that data are accessible on a global scale—an essential element for our system, which caters to a worldwide audience. The crucial aspect of data immortality is also addressed by the IPFS, which ensures that historical information remains available, even in the event of a node failure, and is never lost. In addition, the IPFS maintains data integrity using hashes, ensuring that stored files remain tamper-proof. Integrating the IPFS also reduces blockchain congestion, improves performance, and enhances user privacy, giving them greater control over their data. All in all, this decision promotes interoperability, respects the principle of network neutrality, and ensures that the blockchain remains secure.
**Algorithm 3.** Check Counterfeiting Product

1: **function** CHECKCOUNTERFEITINGPROCESS (*Nnv*)
2: **Input**: T transaction  
▷
transaction of checking of products in blockchain
3: **Output**: S Boolean
▷
State of the product in the blockchain.
4:
      
P1 = getProductIdFromTransaction (T)
5: *List <Product> Products = getValidatedProductFromBlockChain()*
*6:* **for each** product *Pi* in *Products*
**do**

*7:*                
**if**
*Pi = P1*
**then**
8:                 
S ← *true*
9: 
      
**else if** Pi != P1 then
10:
                    
*S*
←*    false*
11:
              
**end if**
12:
        
**end for**
13: Return S
14: **end function**


#### 3.1.3. Application Layer

The application layer, positioned as the highest layer in the architecture, acts as a crucial interface connecting the architecture with external systems, including the smart tourism ecosystem. Its primary role involves receiving user data, conducting necessary processing, and delivering the processed information to customers through a decentralized application. This empowers customers to verify product ownership on a blockchain network before making purchase decisions. The layer exhibits flexibility by offering diverse interaction formats such as a web and a mobile format with an installed verification application, catering to the specific needs of customers and enabling seamless integration with underlying layers. Additionally, it efficiently manages communication with business layer services, promptly handling external user requests and providing timely feedback to ensure optimal system efficiency.

### 3.2. Contributions and Novelty of Approach

The proposed architecture for the craft environment introduces a three-tier model designed to revolutionize the way craft and related industries manage operations and interactions within their environments. This innovative model provides a robust framework that enhances both functionality and user engagement through distinct, purpose-driven layers to combat counterfeiting. Each layer is designed to address specific aspects of system operations, from data collection and storage to processing and user interface, ensuring a complete solution that not only meets today’s technological requirements but also lays the foundations for future advances. This structured approach not only optimizes operational efficiency but also opens up new avenues for the integration and scalability of technologies in the craft sector, representing a significant step forward in the design of intelligent systems.

### 3.3. Experiment and Technical Settings

To better understand the scalability and efficiency of our system, we evaluated the computational complexity associated with the execution and operation of smart contracts and the integration of the IPFS. Using a standard PC configuration highlighted the resource constraints typical of many end-user environments, providing a realistic benchmark for system performance under moderate load.

The hardware configuration of the PC included 8 GB of RAM and an Intel 2.8 GHz processor. These specifications were chosen to ensure sufficient computing power and memory capacity to execute the system efficiently and accurately. The selected PC provided a reliable and capable environment for testing and evaluating the performance of the proposed system. This equipment is used solely for prototype testing the system to assess its performance.

For the development and testing of the decentralized application, we used Ganache as our blockchain for Ethereum development, which allowed for the easy deployment and testing of smart contracts. Solidity was employed as the programming language for writing smart contracts, ensuring secure and efficient transaction management within the system. Additionally, the IPFS was integrated to handle large data storage and retrieval in a decentralized manner, enhancing the system’s performance in terms of speed and reliability when accessing distributed files. This combination of technologies provided a robust framework for our decentralized system, demonstrating significant improvements in security and efficiency over traditional centralized solutions.

The computational complexity of running smart contracts on Ethereum is primarily influenced by their operational logic and the data they process. Smart contracts compiled from Solidity into EVM bytecode have a complexity that can range from O(1) for simple transactions to O(n) or more for operations involving iterative processes over dynamic data structures. Our tests aimed to optimize the efficiency of the contracts by minimizing the number of calculation steps and, consequently, the transaction costs (gas charges).

The IPFS adds another layer of complexity, particularly in terms of data retrieval time, which can vary depending on the network topology and the number of nodes between the data request and the data source. The complexity of the retrieval generally scales with the size of the network and, in decentralized systems, can often be modeled as O(log n), where “n” represents the number of nodes in the network, improving the overall user experience and system performance.

These complexity considerations are crucial to understanding the trade-offs between decentralization, performance, and security in blockchain applications. The configuration of the experiment with a mid-range PC and the detailed analysis of transaction latency and computational load provide valuable insights into the practical implications of deploying such a system in a real environment.

## 4. Experiment Results and Discussions

As part of our research, we reviewed a wide range of works from various fields to support our analysis and enrich our understanding of key concepts. These works, from fields as diverse as engineering, production management, and marketing, provide valuable insights and proven methodologies that prove beneficial to various aspects of our study.

However, it is essential to note that our approach differs from these studies in its exclusive focus on craft products. Despite the wealth of existing literature in these other fields, we found a notable gap in their direct applicability to the craft context. The particularities of craft work, with its traditional techniques and personalized approach, are not explicitly addressed in the work we reviewed.

To improve the effectiveness of the proposed decentralized system, a comparative study was carried out, based on a series of experiments on key parameters such as response time, requests per second, and recovery time. These experimental evaluations were compared with those of a traditional centralized architecture developed in HTML/JS/PHP with a classic central database, specifically defined for this exercise. By analyzing and comparing the results obtained by the two systems, we were able to realize the relative efficiency and performance advantages offered by the decentralized blockchain-based approach.

**Figure 4:**
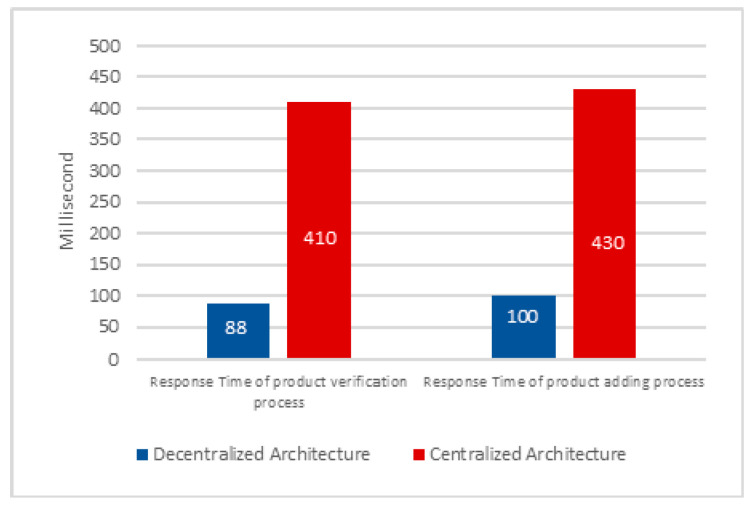
Response time graph of product verification and product adding process.

Figure 4 presents the response time results for two processes: the verification of an artisanal product and the addition of a new product to both centralized and decentralized systems. The blue bar represents the average response time for the verification and addition processes in the case of the centralized system, while the red bar represents the average response time for the decentralized blockchain-based system for the verification and addition processes. By comparing the obtained results, the response time for verification in the centralized system is 366.36% longer than in the decentralized system (410 vs. 88). Similarly, for the adding process, the response time in the centralized system is 330% longer than its decentralized counterpart (430 vs. 100). These results highlight the efficiency of the decentralized blockchain-based approach in handling product verification and addition processes.

**Figure 5:**
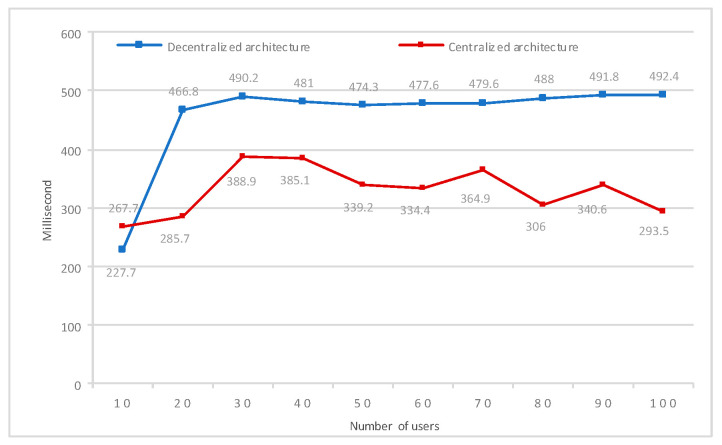
System transaction latency by number of users.

To assess the performance of the two systems, centralized and decentralized, we compared them using the requests per second (RPS) metric, a measure commonly adopted to assess system throughput. Figure 5 shows the results for the verification process of products sent by a variable number of users in both systems. Although the decentralized system demonstrates a superior ability to process a greater number of requests per second, its transaction latency remains approximately 38.21% higher than that of the centralized system. This reveals an important trade-off: while the decentralized system offers benefits in terms of security and autonomy, this architecture results in increased transaction latency. To mitigate this trade-off, it would be possible to explore technical optimizations, such as adjusting the consensus mechanism or using hybrid techniques, which could maintain the benefits of decentralization while reducing transaction latency. This discussion highlights the challenge of reconciling scalability and responsiveness in decentralized systems in order to meet user demands that will be addressed in future work.

**Figure 6:**
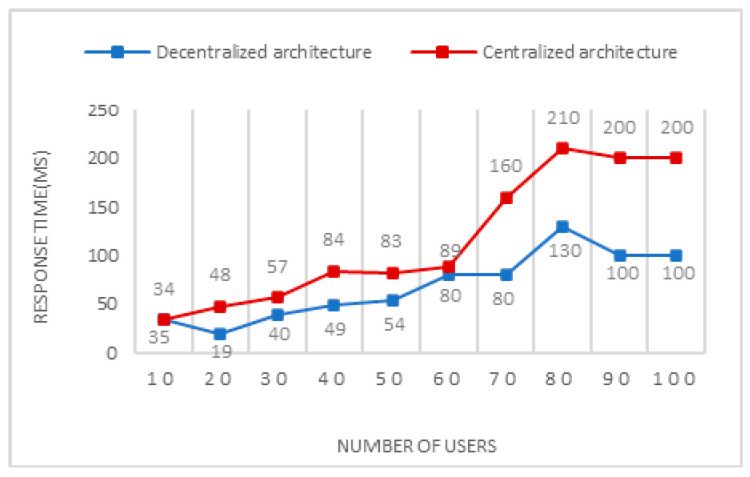
Response time by number of users.

To further evaluate the response time of the decentralized and centralized systems across different user numbers, an experiment was conducted using two distinct test environments: one for a blockchain-based distributed system and the other for a traditional centralized system. Both systems underwent testing with user numbers ranging from 1 to 100. Each system was subjected to 1000 requests, and their response times were measured in milliseconds (MS). The experiment’s results, depicted in Figure 6, demonstrate that the decentralized system built on blockchain technology consistently exhibits lower response times compared to the centralized system, irrespective of the number of users. The decentralized approach achieves a response time of 679 MS, compared to 1365 MS for the centralized approach, highlighting a significant reduction of approximately 50.3% in the response time with the decentralized system. This finding suggests that the decentralized system’s architecture provides improved efficiency and responsiveness, ensuring the quicker processing of user requests and delivering a smoother user experience.

In our approach, we have used only the concept of Proof of Authenticity (PoA) for our decentralized blockchain application, without impacting the operation of the proof of stake (PoS) used in Ethereum. This approach offers advantages in terms of performance and sustainability over other mechanisms, such as Proof of Work (PoW). A comparison is presented in Table 4, highlighting the key differences between these two methods in terms of energy consumption, transaction throughput, speed, scalability, and security:

This comparison illustrates the effectiveness of PoA, particularly in contexts where scalability and integration with technologies such as IoT are crucial. Although security in the PoA relies heavily on the credibility of validators, which can be perceived as a centralization risk, it has proven effective in ensuring fast and economically viable transactions while facilitating the secure exchange of data between platform participants.

- Failover and Recovery Time

Concerning recovery times, the results obtained show significant differences between decentralized and centralized systems. The decentralized blockchain application takes advantage of its intrinsic robustness against total system failure thanks to its distributed nature. Even if some nodes fail, the system retains partial functionality, allowing operations to continue, with full recovery of all nodes possible within 24 h. On the other hand, the centralized system, which is based on a single server, suffers from a total interruption of service in the event of failure of this server. This requires a very low RTO, typically between 4 and 6 h, to minimize the impact on business. Although potentially faster to restore, the vulnerability of the centralized system to a single point of failure presents a higher risk of serious service disruption. As a result, the decentralized approach offers greater resilience and improved business continuity, proving crucial to maintaining business operations and customer confidence.

The results obtained from various experiments clearly demonstrate the superior potential of the decentralized system compared with centralized solutions in protecting the artistic value of craft objects against counterfeiting. This decentralized system has proved to be reliable, efficient, and capable of providing users with an effective means of making informed decisions when buying or selling items. Expected to help preserve the integrity of the craft industry, the system also creates a secure environment for both producers and consumers.

The results also demonstrate superior efficiency, confirming the practicality and effectiveness of the decentralized approach in safeguarding the uniqueness and integrity of artisanal products. Notably, the response time decreases with the decentralized approach and increases with the centralized approach. Similarly, the system transaction latency decreases with the decentralized approach. In addition, the response time as a function of the number of users decreases in the decentralized approach, illustrating the better scalability and adaptability of the system to an increase in demand. These measurements highlight not only the improved operational performance but also the increased efficiency of the decentralized system, reinforcing its relevance and viability for applications in the crafts sector, where preserving the authenticity and quality of works of art is paramount.

Table 5 outlines the parameters and results of various evaluations comparing centralized and decentralized blockchain systems across different metrics and functional aspects, highlighting the benefits and performance enhancements of decentralized systems.

## 5. Impact and Applications of the Proposed System

### 5.1. Economic Impact

From an economic perspective, the adoption of this approach in the craft sector could have a significant impact both locally and globally. Locally, the platform can boost consumer confidence and satisfaction by guaranteeing the authenticity of products through traceability and transparency, which increase sales and potentially enable premium pricing for authenticated products, directly benefiting local artisans and sellers. In addition, by reducing losses from counterfeiting, the platform helps to preserve the incomes of local artisans, thereby retaining more wealth within the community. The implementation and maintenance of this new system architecture could also require the hiring of technical staff, including system managers, data analysts, and support staff, which could help to create jobs in the local technology sector. In addition, artisans using this system can differentiate their products based on verified quality and authenticity, boosting their competitiveness in local and regional markets.

On a global scale, the platform can facilitate the expansion of local craft products into new and more lucrative markets by offering a verified guarantee of authenticity, attracting investment not only in the craft sector but also in the technology that supports it. This can lead to more innovation and development, fostering economic growth. The success of such a platform could also set new standards for product authentication and supply chain transparency, influencing global practice and potentially leading to the adoption of similar technologies in other sectors. By promoting the longevity and sustainability of traditional crafts, the platform supports economic sustainability, with positive long-term effects on global cultural tourism, an important economic sector for many countries.

Furthermore, the integration of blockchain and the Internet of Things (IoT) in the craft sector could profoundly transform local and global economic dynamics by enhancing the transparency, efficiency, and competitiveness of craft products. On a local level, blockchain offers reliable traceability that guarantees the authenticity of craft products, thereby increasing consumer confidence. This transparency enables artisans to justify higher prices for their authenticated products, attracting quality-conscious consumers who are prepared to pay a premium for products whose origin is certified. In this way, sales can increase, while enabling artisans to receive fair remuneration for their work.

The use of blockchain also enables artisans to sell directly to consumers, eliminating intermediaries, thereby reducing distribution costs and increasing the share of profits retained by local producers. This elimination of intermediaries not only supports the economic viability of artisans but also helps to develop the local economy by consolidating a direct sales channel between the producer and the consumer. Blockchain also improves supply chain management by enabling the real-time tracking of materials and finished products. By providing a transparent, tamper-proof record of transactions, artisans can better control the flow of goods, optimize their inventory, reduce waste, and minimize delivery times. This improvement in the supply chain is crucial to meeting production schedules, reducing inventory costs, and satisfying customers, all of which contribute to the stability of craftsmen’s incomes.

The IoT adds a continuous monitoring dimension that optimizes the use of resources. Thanks to IoT sensors, craftsmen can monitor their consumption of raw materials and energy in real time, thereby reducing waste and production costs. This fine-tuned management of resources enables more sustainable and environmentally friendly manufacturing practices to be put in place, a further argument in favor of authentic artisanal products in the eyes of consumers. In addition, the IoT enables direct interaction with customers through personalized marketing strategies based on the analysis of purchasing behavior. The data collected by IoT devices provides valuable information about customer preferences, enabling artisans to better target their offers and strengthen consumer loyalty. This personalized approach boosts sales and helps build lasting relationships between craftspeople and their customers. The IoT also helps to automate administrative tasks such as stock management or order processing, freeing up time for craftspeople to focus more on the creative and innovative aspects of their work. This automation helps to boost productivity while enabling craftspeople to respond quickly to market demands.

In addition, the combination of blockchain and IoT offers simplified access to global markets. Artisans can exhibit their products on a secure digital platform, reaching an international audience without the need for large marketing budgets. This global reach attracts the attention of foreign consumers, retailers, and even investors interested in authentic artisan products. This increased visibility not only helps to diversify markets but also attracts capital into the craft and technology sectors, stimulating innovation and economic development. Finally, by reducing losses due to counterfeiting, this platform protects the income of craftspeople and maintains wealth within local communities. The increased demand for authentic products is also boosting local employment, with a growing need for technical staff to implement and maintain this digital architecture, creating job opportunities for systems managers, data analysts, and technological support staff. By encouraging the use of these cutting-edge technologies, this initiative will enable artisans to differentiate themselves on the basis of authenticity and quality, thereby strengthening their competitiveness.

### 5.2. Socio-Economic Impact

Our system is not just a technical improvement; it also has the potential to transform the socio-economic dynamics within artisanal communities. By ensuring greater traceability and transparency, it can boost consumer confidence, which in turn could stimulate demand for authentic artisanal products. Indeed, consumers are increasingly concerned about the origin and authenticity of the products they buy, and a reliable traceability system could meet this growing demand.

In addition, by facilitating access to wider markets through digital certification, craftspeople could benefit from better remuneration opportunities and increased recognition of their know-how. Digital certification would enable artisanal products to access online sales platforms more securely, giving local artisans global visibility. It could also encourage international consumers to buy authentic products directly from producers, eliminating intermediaries and increasing the income of artisans.

This approach could also play an essential role in preserving cultural traditions. By increasing the visibility and value of craft products in a globalized context, the system would help to keep ancestral techniques and know-how alive. Younger generations of craftspeople would be encouraged to perpetuate these traditions, knowing that their efforts are recognized and valued on an international scale.

The system could also have a positive impact on local development by creating new employment opportunities. The implementation and maintenance of the system would require specific technical skills, creating jobs in areas such as systems management, data analysis, and technical support. These new jobs would help to boost the local economy and improve the living conditions of artisans and their communities.

### 5.3. Potential Use Case

**Figure 7:**
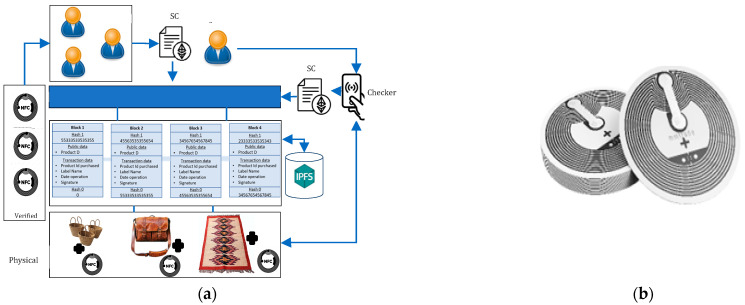
(**a**) Use case in the Moroccan handicraft industry; (**b**) NTG213 type NFC tag.

The system deployed in the Moroccan craft industry, as shown in Figure 7, materializes the proposed approach and illustrates its practical application. This approach ensures the authenticity and quality of craft products. The following is a structured description of how the system is implemented.

Physical layer: Each Moroccan artisanal product is equipped with an NFC tag, specifically an NTG213 type tag, as shown in Figure 7b, which is completely user-programmable before being attached to products. These tags facilitate the initial interaction for product authentication. They enable consumers to quickly check the origin and quality of products by using their smartphone to scan the NFC tag. This type of sensor also has several usability benefits including an immersive digital experience, ease of use, extreme speed, and the highest quality ABS guarantee.

Blockchain layer: The blockchain plays a central role in recording all transactions linked to artisanal products. It securely and immutably stores data such as product origin, production stages, and certifications on the IPFS. This storage method guarantees the permanence and security of the information, making it accessible and verifiable in a decentralized way.

Connectivity and validation: The blockchain network establishes connections between all the participants in the system, thereby facilitating the secure exchange of information. The validators, who are members of the “Chambre Régionales d’Artisanat”, use smart contracts for the process to prove authenticity when adding new data to the blockchain.

Distribution and management of NFC tags: Once the authenticity of the information has been verified by members of the Regional Chambers of Moroccan Crafts, these validators distribute the authenticated NFC tags to the craftsmen. These tags are then affixed to the products, ready for sale, as part of the labeling process for Moroccan artisan brands.

Consumer interaction: End users can verify the authenticity and condition of the Moroccan artisanal product by scanning the NFC tag with their smartphone. During this verification, a dedicated smart contract is executed to check the information stored on the blockchain and to return the results directly to the user. This process ensures total transparency and bolsters consumer confidence in their purchases.

## 6. Challenges of the Proposed System

In our approach, the proposed system is designed to prevent counterfeiting in the craft sector using the Proof of Authenticity consensus algorithm; however, we acknowledge the need for a more in-depth examination of security aspects, particularly concerning the risk of validator centralization. Currently, the system relies on pre-approved validators, such as members of local Artisan Chambers, chosen for their expertise in product authenticity. This setup, however, presents a potential risk of centralization as it depends on the reliability and integrity of these validators.

To enhance security and reduce the risk of centralization, we are considering several improvement pathways. First, we propose expanding the validator pool to include not only Artisan Chambers but also independent experts and recognized institutions within the craft sector. Second, we are exploring a hybrid governance model where the community itself could participate in the assessment and supervision of validators through decentralized rating or voting systems.

Additionally, we plan to implement regular audits and performance evaluations for each validator to ensure a high level of transparency and integrity in the authentication processes. Finally, to meet heightened security requirements, we are investigating the use of advanced cryptographic verification techniques to validate validators’ actions, thus minimizing the reliance on a limited number of actors while maintaining system efficiency.

By incorporating these enhancements, our system aims to balance the specialized expertise required for authenticating craft products with a more decentralized distribution of responsibilities, contributing to greater resilience and broader adoption within the craft sector.

## 7. Conclusions and Future Work

In conclusion, the proposed solution based on the BCoT platform offers a robust and dependable approach to combat counterfeiting in the handcrafted product industry. The implemented system provides an efficient mechanism for verifying the authenticity and status of products, enabling end users to make informed decisions during transactions involving handicrafts. The experimental results of our study clearly demonstrate that our system significantly outperforms traditional solutions in terms of key computational performance indicators such as response time, which is minimized within our system, and the number of requests per second (RPS), which reaches its peak in our case. Moreover, our system demonstrates scalability, adapting effectively to varying numbers of users. These results highlight the outstanding potential of our BCoT-based solution to guarantee the integrity and reliability of handcrafted products while making a significant contribution to the overall improvement of the user experience.

The future prospects for this system are promising. Integrating machine learning technology with all its advantages into the proposed architecture improves the functionality of the proposed system. Subsequently, future work can explore this solution in areas beyond hand-crafted products, where authenticity is of the utmost importance, such as the automotive industry, food products, and others. By leveraging the power of the technologies used in the system, we can extend the benefits of this solution to various industries, guaranteeing the integrity and authenticity of a wide range of products. Future research efforts can focus on improving the scalability and interoperability of the proposed platform, enabling seamless integration with existing supply chain networks and systems. In addition, further efforts can be devoted to exploring advanced security measures and privacy-enhancing techniques, strengthening the system’s resilience against potential threats and vulnerabilities. By following these avenues, we will be able to exploit the full potential of this solution and make it a trusted framework for verifying product authenticity across a wide range of industries.

Moreover, as part of future work, we plan to deploy a real system using high-performance equipment, such as robust machines, which will be installed on-site with artisans and the Chamber of Artisans to thoroughly test the system. In addition, we expect that interoperability with other authentication systems or blockchains could promote the adoption of our solution. Therefore, our future research will focus on exploring this interoperability to extend the scope and usefulness of our system.

### Future Scalar and Energy Improvements

To meet the challenges of scalability and energy efficiency, we are considering several avenues for optimization in future work. Firstly, the use of second-layer solutions, such as sidechains or rollups, would reduce energy consumption and improve scalability without compromising security. These solutions offload part of the processing outside the main chain, thereby reducing the resources required. Next, a hybrid model could be developed, combining centralized elements for non-critical tasks with decentralized elements for authenticity validation. This model would offer a balance between security, energy consumption, and scalability by adapting the distribution of resources according to the criticality of the tasks. Finally, future improvements to the Proof of Authenticity (PoA) concept could be explored, specifically in the craft context. This includes the study of resource-efficient consensus mechanisms, such as optimized variants of Proof of Stake (PoS) or energy-efficient consensus algorithms. These directions aim to strengthen the sustainability of our system while guaranteeing its applicability in real contexts.

## Figures and Tables

**Table 1:** Lists the notations used in the paper.

Acronym	Explanation
UNWTO	United Nations World Tourism Organization
IoT	Internet of Things
BC	Blockchain Technology
BCoT	Blockchain of Things
PoAh	Proof of Authentication
PLM	Product Lifecycle Management
RFID	Radio-Frequency Identification
ITSs	Intelligent Transport Systems
UAVs	Unmanned Aerial Vehicles
NFC	Near Field Communication
GPS	Global Positioning System
WSNs	Wireless Sensor Networks
LEACH	Low Energy Adaptive Clustering Hierarchy
CHs	Cluster Heads
PoS	Proof of Stake
IPFS	Inter Planetary File System
RPS	Requests Per Second
MS	Milliseconds
RT	Recovery Time
RTO	Recovery Time Objectif

**Table 2:** Summary table of the literature.

Technology/Approach	Domaine	Advantages	Weaknesses
RFID [4]	Supply chain industry	Enhances traceability Optimizes stock management Reduces wastage and inefficiencies	Vulnerable to easy replication in packaging and labeling Susceptible to counterfeiting
Blockchain [12]	Chemical manufacturing	Ensures traceability, transparency, integrity Ideal for intellectual property management Decentralized ecosystem for secure transactions	High computational power required. Issues related to data privacy standards Slower transaction validation speeds
Blockchain + IoT + UAV [19]	Intelligent Transport Systems (ITSs)	Real-time traffic data collection Data security and integrity thanks to blockchain Optimize traffic flows, improving road safety, reducing congestion	Need to coordinate different technologies (UAV, blockchain, communication)
Blockchain [22]	Industry 4.0	Transforms transparency, security, and efficiency in manufacturing Enhances sustainable manufacturing and PLM through improved traceability and lifecycle assessments	Can be technologically demanding and costly to implement, especially for small to medium enterprises
IoT and NFC [5]	Craft manufacturing	Facilitates real-time data collection, enhances PLM Improves operational efficiency and business performance	Challenges in data security, interoperability, and standardization
Blockchain and IoT [21]	Textile industry	Advanced traceability systems enhance the integrity of supply chains, foster consumer trust with transparent product histories	Dependence on technological infrastructure, potential privacy concerns

**Table 3:** Symbols used in algorithms.

Symbol	Signification
N_nv_	A set of invalidated nodes in a network.
N_t_	A trusted node that verifies the authenticity of invalid blocks.
N_v_	A set of validated nodes in the network.
T_i_	A set of validated transactions in the network.
P_i_	Transaction for checking the status of a product in the blockchain.
S	The state of product check.

**Table 4:** Comparative analysis of Proof of Work vs. Proof of Authenticity.

Metrics	Proof of Work (PoW)	Proof of Authenticity (PoA)
Energy Consumption	Very high, due to continuous computational effort.	Low, based on identity and reputation.
Transaction Throughput	Low, limited by the time to solve puzzles.	High, no delay due to solving puzzles.
Speed	Slow, confirmation delayed by puzzles.	Fast, no puzzles to solve.
Scalability	Limited, by nature very resource-intensive.	Better, requires less computational power.
Security	Very secure if the network is decentralized.	Depends on the reliability of the validators.

**Table 5:** Control parameters and impact on product processes.

Category	Details	Parameters/Metrics	System Type	Observations/Results
Response Time Evaluation	Verification of artisanal product Addition of a new product	Average response time (verification and addition processes)	Centralized vs. decentralized (blockchain)	A decentralized system shows shorter response times for both processes
Requests Per Second (RPS) Analysis	System throughput evaluation	Requests per second (RPS)	Centralized vs. decentralized	Decentralized architecture outperforms centralized in handling increasing RPS, indicating scalability and efficiency
Response Time Across User Numbers	Testing with user numbers ranging from 1 to 100	Response times in milliseconds (MS)	Distributed (blockchain) vs. centralized	Decentralized system consistently exhibits lower response times regardless of user numbers
Consensus Protocol	Evaluation of proof of authenticity consensus algorithms in tourism industry	Consensus efficiency	Blockchain-based systems	Demonstrates superiority over protocols like PoW (Proof of Work) and PoS (Proof of Stake)
Recovery Time	Evaluation of the recovery time for assessing the resilience of the system in failure	Failover and recovery time	Blockchain-based systems	Proven effectiveness in providing recovery time from a failure

## Data Availability

Data are contained within the article.
